# A Blood Bank Standardized Production of Human Platelet Lysate for Mesenchymal Stromal Cell Expansion: Proteomic Characterization and Biological Effects

**DOI:** 10.3389/fcell.2021.650490

**Published:** 2021-05-14

**Authors:** Andrea Bianchetti, Clizia Chinello, Michele Guindani, Simona Braga, Arabella Neva, Rosanna Verardi, Giovanna Piovani, Lisa Pagani, Gina Lisignoli, Fulvio Magni, Domenico Russo, Camillo Almici

**Affiliations:** ^1^Laboratory for Stem Cells Manipulation and Cryopreservation, Blood Bank, Department of Transfusion Medicine, ASST Spedali Civili of Brescia, Brescia, Italy; ^2^Clinical Proteomics and Metabolomics Unit, Department of Medicine and Surgery, University of Milano-Bicocca, Vedano al Lambro, Italy; ^3^Department of Statistics, University of California, Irvine, Irvine, CA, United States; ^4^Biology and Genetics Division, Department of Molecular and Translational Medicine, University of Brescia, Brescia, Italy; ^5^IRCCS, Istituto Ortopedico Rizzoli, SC Laboratorio di Immunoreumatologia e Rigenerazione Tissutale, Bologna, Italy; ^6^Chair of Hematology, Unit of Blood Diseases and Stem Cell Transplantation, University of Brescia, ASST Spedali Civili of Brescia, Brescia, Italy

**Keywords:** mesenchymal stromal cells, human platelet lysate, growth factors, mass spectrometry, blood banks standards

## Abstract

Human platelet lysate (hPL) is considered a valid substitute to fetal bovine serum (FBS) in the expansion of mesenchymal stromal cells (MSC), and it is commonly produced starting from intermediate side products of whole blood donations. Through freeze–thaw cycles, hPL is highly enriched in chemokines, growth factors, and adhesion and immunologic molecules. Cell therapy protocols, using hPL instead of FBS for the expansion of cells, are approved by regulatory authorities without concerns, and its administration in patients is considered safe. However, published data are fairly difficult to compare, since the production of hPL is highly variable. This study proposes to optimize and standardize the hPL productive process by using instruments, technologies, and quality/safety standards required for blood bank activities and products. The quality and improved selection of the starting material (i.e., the whole blood), together with the improvement of the production process, guarantee a product characterized by higher content and quality of growth factors as well as a reduction in batch-to-batch variability. By increasing the number of freeze/thaw cycles from one (hPL1c) to four (hPL4c), we obtained a favorable effect on the release of growth factors from platelet α granules. Those changes have directly translated into biological effects leading to a decreasing doubling time (DT) of MSC expansion at 7 days (49.41 ± 2.62 vs. 40.61 ± 1.11 h, *p* < 0.001). Furthermore, mass spectrometry (MS)-based evaluation has shown that the proliferative effects of hPL4c are also combined with a lower batch-to-batch variability (10–15 vs. 21–31%) at the proteomic level. In conclusion, we have considered lot-to-lot hPL variability, and by the strict application of blood bank standards, we have obtained a standardized, reproducible, safe, cheap, and ready-to-use product.

## Introduction

Since the first report by Friedenstein et al. (1974), human mesenchymal stromal cells (hMSC) have been described and characterized by minimally accepted criteria (Dominici et al., 2006), and have held promises in different clinical settings (Le Blanc and Pittenger, 2005). Nowadays, hMSC have been proved to be a potentially effective therapy for patients with steroid-resistant acute graft-versus-host disease (GVHD) (Le Blanc et al., 2004, 2008), and they can represent a valid and attractive strategy in the tissue engineering setting for applications in regenerative medicine (Korbling and Estrov, 2003). Despite the exponential growth of clinical cell therapy trials, concerns are emerging, whereby the inherent differences between hMSC sources may create both potentials and limitations in clinical protocols (Le Blanc and Davies, 2018). Also, human platelet lysate (hPL), generally considered an ideal substitute of fetal bovine serum (FBS) in hMSC expansion protocols (Lucarelli et al., 2003; Doucet et al., 2005; Capelli et al., 2007; Hemeda et al., 2014; Muraglia et al., 2014; Shih and Burnouf, 2015; Strandberg et al., 2017), needs to be thoroughly defined in terms of its composition and production process. However, in comparison with FBS, hPL has the advantage to be considered safe for patient administration, and it is accepted by regulatory authorities of cell therapy experimental protocols. Published data on the use of hPL for hMSC expansion confirm a statistically superior effect over FBS (Lucarelli et al., 2003; Doucet et al., 2005; Capelli et al., 2007; Hemeda et al., 2014; Muraglia et al., 2014; Shih and Burnouf, 2015; Strandberg et al., 2017), but they are fairly difficult to compare, due to the many variables (Supplementary Table 1) entering the hPL production process (Burnouf et al., 2016); as a result, hPL is not the common denominator, but rather one of the many factors that could promote clinical effects. Therefore, the major objective of the present study has been to optimize and standardize the hPL production process by defining variables and requirements (Table 1) according to blood bank standards (American Association of Blood Banks [AABB], 2020), Good Practice Guidelines (European Directorate for the Quality of Medicines & HealthCare of the Council of Europe [EDQM], 2020a), and European (European Union [EU], 2020) and national (Centro Nazionale Sangue [CNS], 2020a,b) regulations. The rules define the blood donor selection criteria, the technological standards and protocols for processing and storage, the microbiological safety requirements, and the measures to be adopted for process control and product traceability.

**TABLE 1:** Definition of variables and requirements considered in Brescia Blood Bank hPL production process.

**Phase**	**Variables**	**Description and requirements**
Resources	Production system	Closed loop bags (*closed system*)
	Production monitoring and traceability	Blood bank instruments and software, quality assurance system, work for processes and control points
Source and safety	Starting material	Fresh whole blood
	Infectious disease markers	Serology (HIV 1/2, HBV, HCV, and *Treponema pallidum*) and NAT testing (HIV, HBV, HCV, and WNV)
	Donor selection	Male 18–60 years old
	AB0 group	Group 0 BC in group AB CPP
	Sterility	Fungi, aerobic and anaerobic bacteria
	Irradiation	25–50 Gy
	Residual leukocytes in FFP leukocyte-depleted	<1 × 10^6^/unit (flow cytometric assay)
	Residual leukocytes in PRP leukocyte-depleted	<1 × 10^6^/unit (flow cytometric assay)
	Pooling strategy	25 donors (18 BC and 7 CPP)
Production and characterization	Residual red cells in FFP	<6 × 10^9^/L
	Fibrinogen reduction in FFP	>20%
	Dilution medium	CPP
	Platelet count	1 × 10^6^/μl
	Batch-to-batch no. of platelets	CV% < 10
	hPL batch volume	>1,000 ml
	Batch-to-batch volume variation	CV% < 20
	Platelet lysis	Freeze/thaw
	No. of freeze/thaw cycles	4 cycles
	Lysis efficiency	1 cycle vs. 4 cycles (proteomic and proliferation)
	Freezing temperature	−80°C
	Thawing temperature	+37°C
	Removal of debris (centrifugation)	5,000 *g* for 25 min at 22°C
	Tested cells	3 hMSC at the fourth passage used all along the entire experimental design
	Biochemical analysis	pH, proteins, electrolytes, folate, vitamin B_12_, and glucose
	Growth factors analysis	Mass spectrometric analysis
Storage	Temperature	−80°C
Use	hPL concentration in medium	<20%
	Heparin in culture medium	Minimum dose that prevents medium gelation

*BC, buffy coat; CPP, cryoprecipitate-poor plasma; FFP, fresh frozen plasma; PRP, platelet-rich plasma; hMSC, human mesenchymal stromal cells.*

The production processes of the hPL take place in an aseptic *closed system* from donation to packaging, which guarantees the sterility and safety of the product in all the processing steps. The fibrinogen content in the plasma is reduced with a cryoprecipitation process to prevent the gelling of the hPL medium at the time of use and to minimize the addition of heparin. To improve the release process of growth factors from platelet granules, we have increased the freeze/thaw cycles from one to four. Furthermore, in order to verify the effects of standardization on the quality and reproducibility of the hPL batches, we performed a proteomic characterization of hPL, using mass spectrometry technology, and we tested its biological effects on three different batches of hMSC.

## Materials and Methods

### Selection of Whole Blood Donors

The donors are from the Italian Volunteers Blood Association (AVIS) and they regularly donate to the Blood Bank of the ASST Spedali Civili of Brescia, according to Italian national legislation (Centro Nazionale Sangue [CNS], 2020a,b). Donations are voluntary and free. Since the plasma from polytransfused donors and multiparous women may contain antibodies against human leukocyte antigens (HLA) and human neutrophil antigens (HNA), we have decided to exclude their donations for hPL production. For organizational reasons, we have limited our selection to non-transfused males between the ages of 18 and 60, negative in the screening for communicable diseases by serology (HIV 1/2, HBV, HCV, and *Treponema pallidum*) and NAT tests (HIV, HBV, HCV, and WNV) (American Association of Blood Banks [AABB], 2020; Centro Nazionale Sangue [CNS], 2020a,b; European Directorate for the Quality of Medicines & HealthCare of the Council of Europe [EDQM], 2020a; European Union [EU], 2020). It was shown that hMSC do not express or upregulate ABO blood group antigens and that hPL does not exert any detrimental effect on proliferation, regardless of blood group (Moll et al., 2014). However, in order to avoid potential immune activation due to antigens or isoagglutinins and to limit hemolysis during production, we have decided to use group 0 buffy coat and group AB plasma. We used the intermediate by-products of whole blood donations processed within 24 h of collection, which were not necessary for clinical needs.

### hPL Productive Process

**FIGURE 1:**
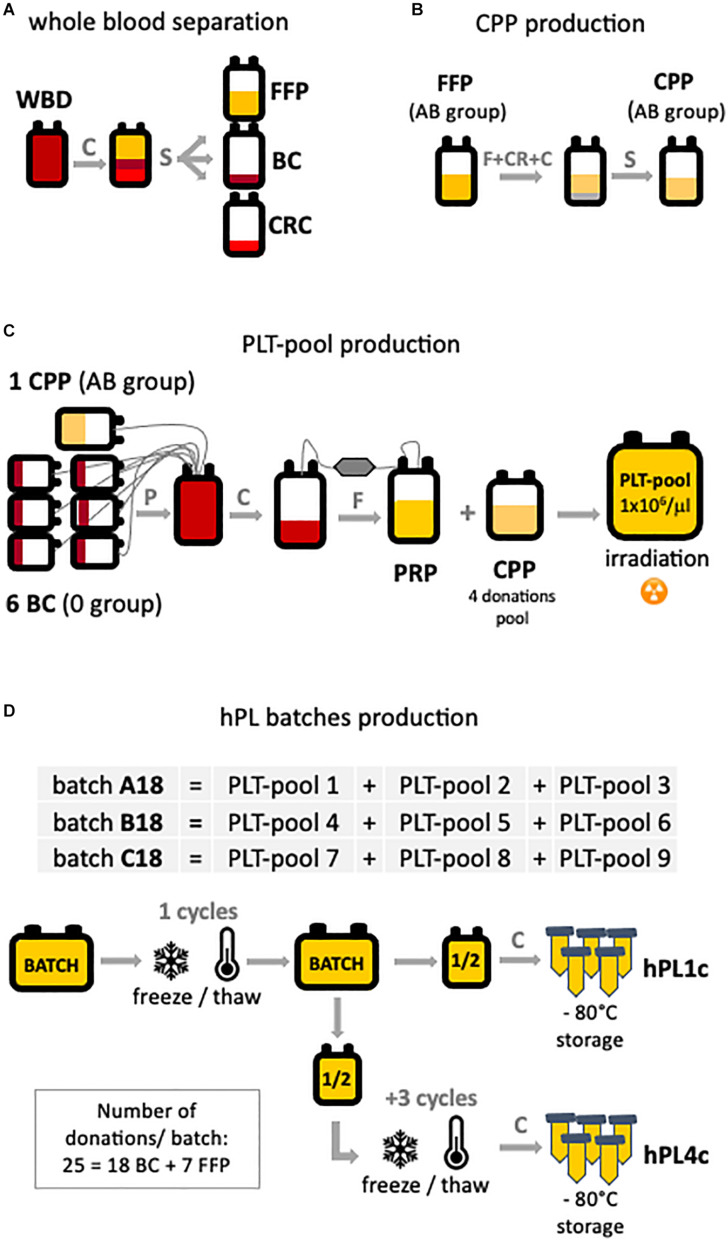
hPL production scheme. The main production steps are highlighted: **(A)** starting from automatic separation of whole blood donation (WBD) in fresh frozen plasma (FFP), buffy coat (BC), and concentrated red cell (CRC) units. **(B)** Cryoprecipitate-poor plasma (CPP) is obtained by removing the cryoglobulin fraction of the plasma from FPP. **(C)** Platelet-rich plasma (PRP) is the product of the processing of six BC and one CPP. To obtain the platelet pool (PLT pool), platelet concentration was diluted to 1 × 10^6^ PLT/μl with CPP. **(D)** Three batches (A–B–C18) of human platelet lysate (hPL) were produced. Each batch is the result of three PLT pools assembled and subjected to one or four freeze/thaw cycles. For each batch, both hPL with one freeze–thaw cycle (hPL1c) and hPL with four freeze–thaw cycles (hPL4c) were produced. After debris removal, hPL was sterile aliquoted and stored at –80°C until use. C, centrifugation; CR, cryoprecipitation; F, filtration; P, pooling; S, separation.

By taking advantage of the Blood Bank of the ASST Spedali Civili of Brescia, we had the opportunity to apply to the production of hPL instruments and technologies (Supplementary Figure 1) quality and safety requirements routinely used by blood banks in the processing of blood products. By automatic whole blood separation (Fresenius Kabi), fresh frozen plasma (FFP), buffy coat (BC), and red blood cell (RBC) units were derived (Figure 1A). FFP was processed to obtain cryoprecipitate-poor plasma (CPP) used in platelet pool (PLT pool) production. Group AB FFP was filtered for leukocyte depletion, rapidly frozen (−80°C, <45 min, PlasmaFrost ITeM, Angelantoni Life Science), and then allowed to thaw overnight in a monitored blood bank refrigerator (+4°C). The morning after, FFP was centrifuged on high speed (3,000 *g*, 15 min, +4°C, Heraeus Cryofuge 6000i) to precipitate and remove the cryoglobulin fraction of plasma (Figure 1B). To produce every single PLT pool (Figure 1C), at maximum 24 h time-lapse from collection, six group 0 BC and one unit of group AB CPP were assembled (Teruflex BP-KIT with Imugard III-S PL, Terumo) and gently centrifuged (340 *g*, 6 min, +22°C, Heraeus Cryofuge 6000i) to obtain platelet-rich plasma (PRP) in-line filtered for leukocyte depletion in a satellite bag. Afterward, PRP was diluted to a platelet concentration of 1.0 × 10^6^/μl with CPP (pool of four donations) and 25–50 Gy irradiated, according to the PLT pool protocol, to reduce the viability of any residual leukocytes. Residual leukocytes were flow cytometry counted (Kit Leucocount, FACSCanto II, BD).

Three hPL batches (A–B–C18) were manufactured by assembling three different PLT pools for each batch (Figure 1D). Assembled pools were rapidly frozen (−80°C, <45 min, PlasmaFrost ITeM, Angelantoni Life Science), stored at −80°C for 24 h in a monitored blood bank freezer, and then thawed at +37°C for 25 min (DH8 Plasma Thawing System, Helmer) to cause platelets’ lysis. The hPL was split in half into two bags. After centrifugation (5,000 *g*, 25 min, +22°C) for the removal of platelet bodies or debris, half of the product hPL (hPL1c) was aliquoted into 50 ml tubes (BD Falcon conical tubes) and stored at −80 ± 10°C until use. In order to improve the releasing process of growth factors, half of the hPL product further underwent three freeze–thaw cycles (hPL4c), and after the removal of debris by centrifugation, it was aliquoted and stored at −80°C as the one-cycle product. The testing of hPL sterility for fungi and aerobic and anaerobic bacteria resulted negative. The described process allowed to produce batches of hPL with a volume greater than 1,000 ml.

### hPL Biochemical Analysis

Three hPL batches at four freeze–thaw cycles (hPL4c A–B–C18) were analyzed for their fibrinogen content (ACL TOP 700 LAS, Instrumentation Laboratory Werfen, Milan, Italy), biochemical compositions (Roche Cobas 8000, Risch-Rotkreuz, Switzerland), protein content by electrophoresis (SEBIA Italia s.r.l., Bagno a Ripoli, Firenze, Italy), and hemoglobin (HemoCue, Ängelholm, Sweden) in comparison with a mix of three lots of commercial FBS (Sigma-Aldrich, Darmstadt, Germany).

### Proteomics Evaluation of hPL Batches by nLC-ESI MS/MS

Proteomic profiles of all the batches of hPL both at one and four freeze/thaw cycles (hPL1c and hPL4c, respectively) and the related CPP, belonging to three different group of donors (groups A, B, and C; *n* = 18 for each group), were evaluated by nano LC-ESI MS/MS.

Before the mass spectrometric analysis, the nine different samples were albumin- and IgG-depleted using the PROTIA Depletion kit (Sigma-Aldrich) and trypsinized (Re et al., 2019). Briefly, 50 μl of sample derivative diluted in 50 μl of equilibration buffer was loaded onto the immunodepletion column, already equilibrated, and incubated for two consecutive times. The twice-depleted specimen was then collected by centrifugation and combined with a final washing step of 125 μl of equilibration buffer. All the eluted fractions were pooled and the protein concentration was determined using bicinchoninic acid assay on microplates (Pierce-Thermo Fisher Scientific). The enzymatic digestion protocol was based on the filter-aided sample preparation (FASP) strategy. In particular, a volume corresponding to 300 μg of proteins for each sample was diluted with ammonium bicarbonate 50 mM and denaturated by dithiothreitol (20 mM DTT; 95°C for 5 min). After disulfide bond reduction, samples were transferred into the ultrafiltration units (Amicon Ultra-0.5 ml 30 kDa, Millipore). FASP digestion was performed (Chinello et al., 2019). Protein digestion was performed overnight at 37°C adding trypsin. Digestion was stopped by acidification by adding formic acid till 0.1% v/v. Tryptic peptide mixtures were concentrated by a vacuum centrifuge and their concentrations were determined using a NanoDrop^TM^ spectrophotometer (Thermo Fisher Scientific).

A volume of sample corresponding to about 1 μg of peptide mixtures was injected into the nanoRSLC system for UHPLC separation (Thermo Fisher Scientific) and analyzed by an online coupled tandem mass spectrometer Impact HDTM UHR-QqToF (Bruker Daltonics) (Bosisio et al., 2020). Three technical replicates for each sample were run in order to take into account and reduce the variability associated with the label-free relative quantification approach. Data elaboration including calibration was performed through DataAnalysis^TM^ v.4.1 Sp4 (Bruker Daltonics, Germany).

Protein identification and quantification were carried out using a proteomic platform based on PEAKS Studio X+ ver. 10.5 (Bioinformatics Solutions Inc., Waterloo, ON, United States). Three LC-MS/MS runs were loaded for each sample. An in-house-constructed UniProt’s reference database of *Homo sapiens* (accessed June 2018, 557,713 sequences; 200,130,199 residues) was combined with a decoy database and implemented in the software. Only confidently identified peptide features [false discovery rate (FDR) ≤1%] were used for the estimation of the related signal intensity. The normalization of the areas under the curve of the extracted ion chromatograms was performed using TIC (total ion current). Proteins with less than two unique peptides were discarded from the analysis. Only peptides having a quality factor ≥5 were considered for the relative quantification. The non-parametric test integrated in PEAKSQ was used to evaluate the significance of the comparisons. A minimum significance of 13 (*p* value ≤ 0.05) and a minimum fold change of 1.5 calculated selecting the three most abundant unique peptides were set. Three different levels of stringency in the peptide filtering of the data were established based on the specific distribution of the ratios for the dataset: (i) low stringency criteria (LSC): Ave-Area ≥5E4, peptide ID count: 0, confidently detected sample: 0; (ii) middle stringency criteria (MSC): Ave-Area ≥1E5, peptide ID count: 0, confidently detected sample: 0; and (iii) high stringency criteria (HSC): Ave-Area ≥5E5, peptide ID count: 1, confidently detected sample: 1.

### Bone Marrow hMSC Batch Production

Bone marrow (BM) cells are obtained from bag washouts of scheduled BM donation, after informed consent from the donors. Nucleated cells are recovered by two washings of the filters’ and bags’ residues with 150 ml PBS (Sigma-Aldrich). Mononuclear cells are recovered by gradient separation (Lympholyte-H, Biosera) (400 *g*, 30 min, +22°C). Viable cells are counted [Trucount tubes, 7-amino-actinomycin D (7-AAD) FACS Canto II, BD] and seeded (3.5 × 10^5^ cells/cm^2^) in plastic culture flasks (SPL Life Sciences) in Iscove’s medium (Sigma-Aldrich) containing penicillin 100 U/ml–streptomycin 100 μg/ml (Sigma-Aldrich), 2 mM L-glutamine (Sigma-Aldrich), 2.5 μg/ml Fungizone (Sigma-Aldrich), and 10% FBS. Non-adherent cells are removed after 48–72 h (+37°C, 5% CO_2_) and the fresh medium was changed every 72 h thereafter. At confluent growth, adherent cells are detached by trypsin 0.25%–EDTA 0.02% (Sigma-Aldrich) treatment and replated at 4 × 10^3^ cells/cm^2^ in the same medium conditions. At each passage, hMSC (5 × 10^5^ cells/100 μl) antigenic profile is determined (FACSCanto II, BD) by using a four-color combination (CD45-Pe-Cy7, CD73-PE, CD90-APC, CD105-PerCPCy5.5) and single monoclonal antibodies (CD34-PE, CD14-FITC, and CD19-PE). The different tests, all along the entire experimental design, have been repeated with three hMSC batches (hMSC1–2–3).

### Effect of hPL Concentration

To test the most effective concentration of hPL4c in hMSC expansion, three different hMSC batches (hMSC1–2–3 at the fourth passage) have been expanded in a medium containing scalar concentrations of hPL4c (A–B–C18) from 0.6 to 20%. Doubling times (DTs) at 4, 7, and 10 days of culture are compared at different hPL concentrations.

### Effect of Heparin Concentration

Since in the literature hPL is mainly used at 5% in culture medium, we looked for the minimum concentration of heparin to be added to the medium based on this percentage. The same hMSC batches (hMSC1–2–3 at the fourth passage) have been cultured in a medium containing 5% hPL4c (A–B–C18) in the presence of standard 2 IU/ml sodium heparin in comparison with decreasing amounts of sodium heparin (1.2, 0.6, 0.3 IU/ml) and a heparin-free condition. DTs at 4, 7, and 10 days of culture are compared in the different medium conditions.

### hPL Effect on hMSC Expansion

The biological effects of hPL at one and four freeze/thaw cycles (hPL1c and hPL4c, respectively) have been tested in comparison to a mix (FBS mix) of three commercially available batches of FBS (Sigma-Aldrich), as well as to hPL A17, as previously characterized and tested (Tran et al., 2019). Briefly, hMSC at the fourth passage (p4) are seeded at 4 × 10^3^ cells/cm^2^ in plastic culture flasks in Iscove’s medium containing penicillin 100 U/ml–streptomycin 100 μg/ml, 2 mM L-glutamine, and 2.5 μg/ml Fungizone (Sigma-Aldrich) in the presence of either 10% FBS mix, 5% hPL1c, 5% hPL4c, or 5% hPL A17. Three different batches (A–B–C18) of hPL1c and hPL4c have been tested. Sodium heparin (0.6 IU/ml) (Veracer) has been added to the medium containing hPL to prevent gellification. The results have been generated in triplicate, performed at different time frames, and repeated with three different hMSC batches (hMSC1–2–3). Half of the hMSC were sacrificed at 4 days and the rest at 7 days of culture. The cells were trypsinized, washed, and resuspended in PBS solution (5% FBS). Cell count and viability were performed in Trucount tubes (BD Biosciences) by the addition of 7-AAD (BD Biosciences) and analyzed by flow cytometry (FACSCanto II, BD Bioscience). hMSC expansion at 4 and 7 days has been calculated as DT expressed in hours^1^.

### Statistical Analysis

Descriptive statistics and graphical displays, including histograms and boxplots, were obtained prior to all inferential analyses to evaluate the appropriateness of distributional assumptions and for visual assessment. Throughout the manuscript, all measurements are reported as mean ± standard deviation (SD). The comparisons between measurements on two culture conditions were assessed by the Welch two-sample *t* test, whereas comparisons among multiple (>2) culture conditions were assessed using one-way ANOVA. To adjust for multiplicity in the ANOVA, a Tukey’s HSD procedure was employed for all pairwise comparisons. All tests were two-sided with a significance level set at 5% (0.05). Whenever applicable, the Benjamini–Hochberg FDR procedure was employed for *p* value adjustment. All the analyses were performed using the freely available language and environment for statistical computing and graphics, R, version 4.0.2^2^.

## Results

### Biochemical Analysis of hPL

The biochemical compositions of the three hPL4c batches that were partially fibrinogen-depleted during the production phase turned out to be overlapping, but as expected, they were different in comparison with the FBS mix (Supplementary Table 2). In more details, fibrinogen ranged from 0.173 to 0.191 g/dl in hPL, with a 31% depletion range, while it was undetectable in the FBS mix. Mechanical methods based on the intentional formation of hydrogels in the hPL medium lead to reducing the initial concentration of fibrinogen by over a thousand folds (Laner-Plamberger et al., 2015). As expected, the removal of the cryoglobulin fraction of the plasma demonstrated lower efficiency. However, with a simple method and without adding additives, we obtained fibrinogen concentrations slightly lower than the values published for hPL with similar characteristics (Shih and Burnouf, 2015; Burnouf et al., 2016). The protein content in hPL, ranging from 5.61 to 6.13 g/dl, was found to be comparable to that of other preparations (Shih and Burnouf, 2015; Burnouf et al., 2016). By chemical analysis, glucose resulted to be significantly higher in hPL in comparison with the FBS mix (average hPL 307.6 vs. 70.1 mg/dl). This result is probably attributable to the additives present in the bags used for whole blood donations, while folate resulted to be lower in hPL compared with the FBS mix (3.6 vs. 6.5 ng/ml). Finally, Ca^++^, Na^+^, K^+^, Cl^–^, P, Mg, and vitamin B_12_ have similar concentrations.

### hPL Mass Spectrometry Analysis and Proteomic Batch-to-Batch Variability

An LC-MS/MS label-free strategy was applied to compare “proteomically” the different preparations of hPL (hPL1c and hPL4c) together with the related CPP counterparts and to measure proteomic batch-to-batch variability. Therefore, the same three batches derived from 25 donors (A–B–C18) used for testing the effect of hPL on hMSC expansion were relatively quantified and characterized by an MS-based proteomic approach. Data were filtered with three increasing levels of stringency – low, middle, and high – as detailed before.

**FIGURE 2:**
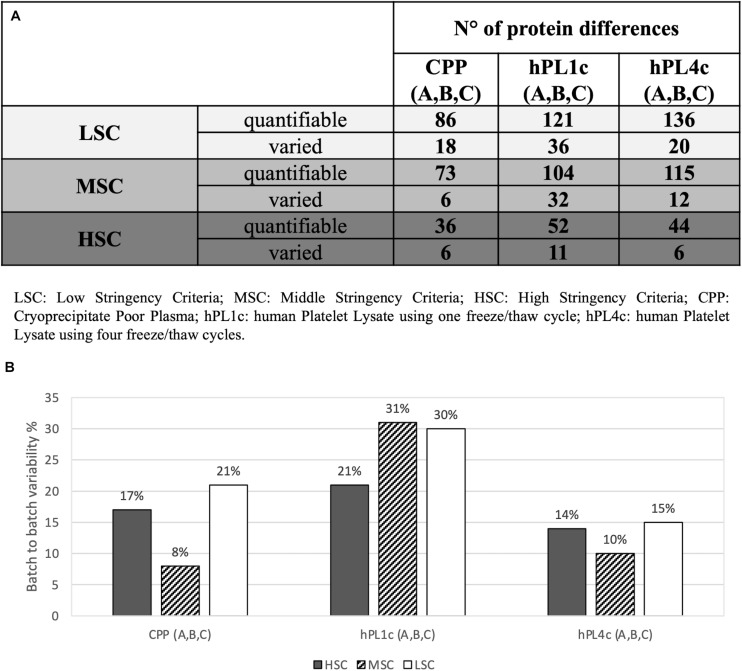
Proteomic batch-to-batch variability in hPL1c and hPL4c and in CPP. Table and graphics report the extent of proteomic variability between different batches (A–C) for the three preparations (hPL1c, hPL4c, CPP). PEAKS Studio X+ platform was applied on outcomes from label-free relative quantification based on nLC-ESI-MS/MS. The variability was considered taking into account three different levels of stringency (LSC, low stringency criteria; MSC, middle stringency criteria; HSC, high stringency criteria) in order to make the results more reliable and to exclude any possible effect derived from data filtering. For each condition, the variability was calculated as the ratio between the proteins significantly varied between three different batches (A–C) (*p* < 0.05, fold change ≥1.5, at least two unique peptides/protein) over the numbers of quantifiable proteins (significance = 0, at least two unique peptides/protein); **(A)** table with the number of quantifiable proteins and batch-to-batch significant differences for all the samples and the stringency levels; **(B)** graphic illustrating batch-to-batch variability in percentage (significantly varied over quantifiable proteins) for all the samples and the stringency levels. CPP, cryoprecipitate-poor plasma; hPL1c, human platelet lysate using one freeze/thaw cycle; hPL4c, human platelet lysate using four freeze/thaw cycles.

**FIGURE 3:**
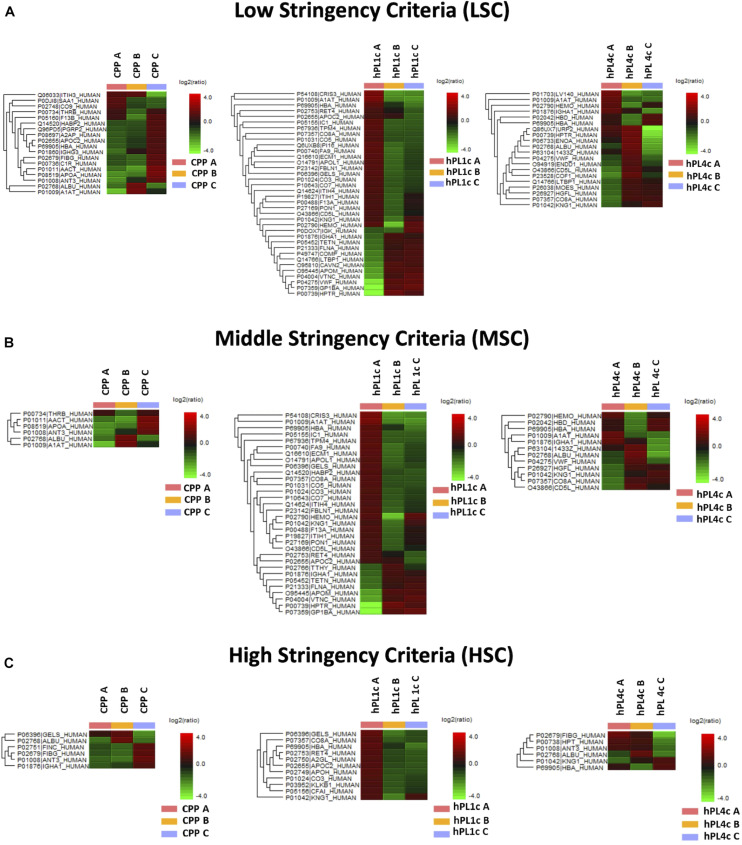
Proteome differences for batch-to-batch variability in CPP, hPL1c, and hPL4c. Heatmaps of significant protein differences (*p* < 0.05, fold change ≥1.5, at least two unique peptides/protein) between three different batches (A–C) for CPP, hPL1c, and hPLC4c applying three levels of stringency for peptide filtering in PEAKS Studio X+. UniProt names and the relative normalized abundance ratio of the protein differences are illustrated and plotted in the heatmaps. CPP, cryoprecipitate-poor plasma; hPL1c, human platelet lysate using one freeze/thaw cycle; hPL4c, human platelet lysate using four freeze/thaw cycles. **(A)** Low stringency criteria (LSC): Ave-Area ≥5E4, peptide ID count: 0, confidently detected sample: 0; **(B)** Middle stringency criteria (MSC): Ave-Area ≥1E5, peptide ID count: 0, confidently detected sample: 0; **(C)** High stringency criteria (HSC): Ave-Area ≥5E5, peptide ID count: 1, confidently detected sample: 1. A higher number of proteins highlights a greater variability. As shown, independently from the applied conditions, hPL1c shows a wider variability compared with hPL4c and with its starting point CPP.

Independently from the set parameters, the hPL4c has always shown lower batch-to-batch variability than hPL1c in terms of the number of proteins varied between batches vs. the number of quantifiable proteins (Figure 2A). Applying high stringency criteria, batch-to-batch variability was 17, 21, and 14% for PPP, hPL1c, and hPL4c, respectively (Figure 2B), suggesting that the improvement in cell culture expansion is combined with an increase in the stability of the preparation for hPL4c. Among the differentially abundant proteins observed across the three batches of hPL4c, only three proteins varied across the high, middle, or low data filtering conditions (ALBU_HUMAN, HBA_HUMAN, and KNG1_HUMAN). As a comparison, seven proteins were found to vary for hPL1c (GELS_HUMAN, HBA_HUMAN, KNG1_HUMAN, CO8A_HUMAN, RET4_HUMAN, APOC2_HUMAN, and CO3_HUMAN) and two for PPP (ALBU_HUMAN, ANT3_HUMAN) (Figure 3). Proteomic analysis of the two different human lysate samples hPL1c and hPL4c allowed to identify a total of 383 different protein IDs showing at least one significant and unique peptide (FDR < 1%); 331 of them, corresponding to 86% of the total, were common between the two diverse protocols of preparations. Moreover, eight proteins were present in hPL1c and 44 were present in hPL4c (Supplementary Table 3). Among the proteins that were only detectable in hPL4c, three were recognized as growth factors related to the regulation of insulin-like growth factor (IGF) transport and uptake (SERPIND1, PDIA6, PRKCSH).

### hMSC Batch Preparation

We have produced hMSC in large batches from three BM donations (hMSC1–2–3), in order to use each single one along the entire evaluation process. The second-passage hMSC have been stored in liquid nitrogen at the concentration of 0.5 × 10^6^ cells/vial until subsequent use. Up to the third passage, hMSC were cultivated in 10% FBS medium in order to ensure equal culture conditions for subsequent experiments in the different culture media (Supplementary Figure 2A). Before freezing, each hMSC batch was characterized for antigenic profile and differentiation potential, according to minimal accepted criteria (Dominici et al., 2006). The hMSC antigenic profile was proved to be positive (>99%) for CD105, CD73, and CD90 and negative for CD45, CD34, CD14, and CD19 (Supplementary Figure 2B). Osteogenic, adipogenic, and chondrogenic differentiation potentials of hMSC have been determined by using specific differentiating media (Supplementary Figure 2C).

### Effect of hPL Concentration

In order to identify the most effective hPL4c concentration for hMSC expansion, we have progressively doubled the concentration of hPL4c (A–B–C18) from 0.6 to 20%, keeping the sodium heparin concentration fixed at 0.6 IU/ml (Supplementary Table 4). In the expansion cultures containing 0.6% hPL4c, cellular proliferation did not proceed for some cultures. The culture medium with 5% hPL4c proved to be the best condition for 10 days of hMSC expansion (average DT 69.37 ± 1.96 h), compared with 1.25% hPL4c (average DT 112.77 ± 6.37 h), 2.5% hPL4c (average DT 86.57 ± 3.45 h), and 10% hPL4c (average DT 71.30 ± 0.75 h). With 10 and 20% hPL4c, the culture medium gelled, and with 20% hPL4c, it was not possible to recover the cells at the time of counting. When using 10% hPL4c with 2 IU/ml heparin, the medium did not gel (average DT 86.30 ± 2.30 h) and also when using 20% hPL4c with 2 IU/ml heparin (average DT 84.17 ± 2.83 h).

### Effect of Heparin Concentration

Considering that the use of hPL for hMSC expansion requires sodium heparin to be added to the final medium in order to prevent gelling and that, in our hPL4c production process, we reduced the cryoprecipitate fraction of plasma, we have investigated if it were possible to avoid or at least reduce the amount of sodium heparin to be added to the final medium, containing 5% hPL4c. For the three hPL4c batches tested, 0.6 IU/ml was shown to be the lowest concentration of sodium heparin needed to prevent gelling of the culture medium. At 10 days of culture, the average DT was 67.84 ± 5.76 h with 0.6 IU/ml sodium heparin, 80.00 ± 8.54 h with 2 IU/ml sodium heparin, and 72.48 ± 7.66 h with sodium heparin-free medium (0.6 vs. 2 IU/ml, *p* = 0.0161). The heparin-free and 0.3 IU/ml heparin culture medium gelled, but it was still possible to recover the cells by trypsin treatment (Supplementary Table 4).

### hMSC Expansion (FBS vs. hPL1c vs. hPL4c)

**FIGURE 4:**
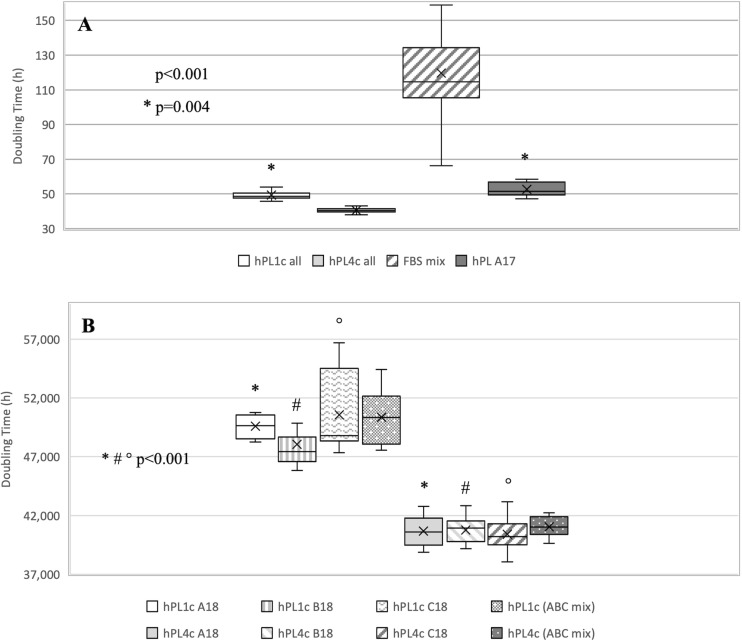
Boxplot of hMSC expansion at 7 days of culture in the presence of FBS vs. hPL1c and hPL4c. At the start of the expansion assay (day 0), 4,000/cm^2^ hMSC are seeded in the presence of 10% FBS mix and 5% of three different batches of hPL1c and hPL4c. Cell proliferation is expressed as doubling time (hours). *X* indicates sample mean. **(A)** The results of all experiments regardless of hMSC and hPL batches used. hPL A17: control-hPL batch previously produced and characterized (18 donations of BC, one freeze/thaw cycle, plasma without removal of fibrinogen, sodium heparin 2 IU/ml). hPL4c (mean DT 40.61 ± 1.11 h) shows an expansion effect on hMSC higher than the hPL1c (mean DT 49.41 ± 2.62 h, *p* < 0.001), hPL A17 (mean DT 52.66 ± 3.90 h, *p* < 0.001), and the FBS mix (mean DT 119.70 ± 32.36 h, *p* < 0.001). The proliferative effect of hPL1c is comparable to that of hPL A17 (*p* = 0.004), but it is higher than that of the FBS mix (*p* < 0.001). Medians: hPL1c all 48.60, hPL4c all 40.56, FBS mix 114.70, hPL A17 51.58. **(B)** The results for each hPL1c and hPL4c batch and for the mix (batches A + B + C) at one and four cycles. hPL4c demonstrates a greater proliferative effect than hPL1c even considering single batches (*p* < 0.001). No significant differences in DT were measured between the hPL batches at one and four cycles, respectively. hPL4c mix (mean DT 41.03 ± 0.83 h) showed no positive or negative effect compared with the single batches. Medians: hPL1c (ABC mix) 50.35 and hPL4c (ABC mix) 41.02.

**FIGURE 5:**
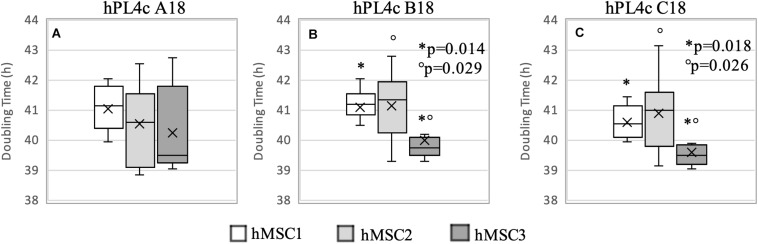
Boxplot of hMSC expansion at 7 days of culture in the presence of hPL4c. At the start of the expansion assay (day 0), 4,000/cm^2^ hMSC are seeded in the presence of 5% of three different batches of hPL4c. Cell proliferation is expressed as doubling time (hours). *X* indicates sample mean. **(A)** Effect of hPL4c A18 on three different hMSC batches. hPL4c A18 has shown no differences in DT for the three hMSC batches. **(B)** Effect of hPL4c B18 on three different hMSC batches. hPL4c B18 has shown higher proliferative effect on hMSC3 compared with the other hMSC batches (hMSC1 vs. hMSC3, *p* = 0.014, and hMSC2 vs. hMSC3, *p* = 0.029). **(C)** Effect of hPL4c C18 on three different hMSC batches. hPL4c C18 has shown higher proliferative effect on hMSC3 compared with the other hMSC batches (hMSC1 vs. hMSC3, *p* = 0.018, and hMSC2 vs. hMSC3, *p* = 0.026).

Regardless of the hPL and hMSC batches being used, at 4 days of culture, hPL4c showed an increased proliferative effect (mean DT 36.06 ± 4.03 h) compared with hPL1c (mean DT 39.94 ± 1.13 h, *p* < 0.001), hPL A17 (mean DT 45.71 ± 4.48 h, *p* < 0.001), and the FBS mix (mean DT 66.73 ± 6.93 h, *p* < 0.001) (Supplementary Figure 3A). Even considering the results of each single batch at 4 days of culture, the higher proliferation rate is confirmed when using hPL4c vs. hPL1c (A18 *p* < 0.001, B18 *p* = 0.01, C18 *p* < 0.001) (Supplementary Figure 3B). Even at 7 days of culture, hPL4c (mean DT 40.61 ± 1.11 h) confirms an expansion effect on hMSC higher than the hPL1c (mean DT 49.41 ± 2.62 h, *p* < 0.001), hPL A17 (mean DT 52.66 ± 3.90 h, *p* < 0.001), and the FBS mix (mean DT 119.70 ± 32.36 h, *p* < 0.001). The proliferative effect of hPL1c is comparable to that of hPL A17, our previous production with one freeze/thaw cycle (*p* = 0.004), but it is higher than that of the FBS mix (*p* < 0.001, Figure 4A). The greater proliferative thrust of hPL4c, compared with hPL1c, is also confirmed by considering the single hPL batches (*p* < 0.001, Figure 4B). No significant differences in DT were measured between the hPL batches at one and four cycles, respectively. However, a higher coefficient of variation between batches can be noted in hPL1c compared with hPL4c (5.39 vs. 2.74 CV%). In addition, hMSC cultured with a mix of the three hPL1c (mean DT 50.36 ± 2.15 h) had a lower proliferative effect than the single hPL1c B18 batch (*p* = 0.045); however, this last result needs to be verified with a larger number of measurements. On the other hand, the hPL4c mix (mean DT 41.03 ± 0.83 h) showed no positive or negative effect compared with the single batches (Figure 4B). We also tested the proliferative effect of each single hPL4c at 7 days of culture on three different hMSC batches. While hPL4c A18 has shown no differences in DT for the three hMSC batches (Figure 5A), the hPL4c B18 (Figure 5B) and C18 (Figure 5C) have shown higher proliferative effect on hMSC3 compared with the other hMSC batches (B18 hMSC1 vs. hMSC3, *p* = 0.014, and hMSC2 vs. hMSC3, *p* = 0.029; C18 hMSC1 vs. hMSC3, *p* = 0.018, and hMSC2 vs. hMSC3, *p* = 0.026).

Overall, the hMSC3 showed reduced DT with all hPL4c batches (mean DT 39.97 ± 1.12 h, median 39.55, CV% 2.81). However, no significant differences were observed between the hPL4c batches when comparing the DT within each hMSC (Figure 5), all resulting <50 h.

### Variables and Requirements for hPL Release

Scientific organizations and national entities have been committed to the standardization of the processes and they have released criteria for the harmonization of raw materials such as hPL (Strunk et al., 2018; Biebak et al., 2019; Henschler et al., 2019; European Directorate for the Quality of Medicines & HealthCare of the Council of Europe [EDQM], 2020b; Schallmoser et al., 2020). Although the blood bank standards and procedures (American Association of Blood Banks [AABB], 2020; European Directorate for the Quality of Medicines & HealthCare of the Council of Europe [EDQM], 2020a; European Union [EU], 2020) are the key reference in blood management, we have defined specific requirements for the production of hPL (Table 1). According to the European guidelines (European Directorate for the Quality of Medicines & HealthCare of the Council of Europe [EDQM], 2020a), we have limited hPL residual leukocyte content by irradiation and filtration, far below the accepted value of 1 × 10^6^/unit. Our production process involves batches with 18 BC units to reduce the variability between hPL batches without neglecting the risk of infection. Platelets, diluted in CPP (1 × 10^6^/μl), were subjected to lysis to obtain more than 1,000 ml of hPL. Although not always validated (Strunk et al., 2018; Biebak et al., 2019; Henschler et al., 2019), we have used the freeze/thaw method as the lysis process. The cycle number efficiency was evaluated by comparing proteomic analysis and the proliferative effect of hPL1c vs. hPL4c. Based on the different hPL variables and requirements, we established the release standards and the minimum tolerable batch-to-batch variability (Table 2). Lastly, we have also considered hPL4c stability after 6, 12, and 24 months of storage at −80°C (Supplementary Figure 4). We have verified that the proliferative efficacy of hPL4c batches (A–B–C18), tested on hMSC2, remains stable after 24 months from production (mean DT 40.80 ± 1.71 h).

**TABLE 2:** Standards for hPL release.

**Quality control**	**Parameter**	**Range for release**	**Batch-to-batch variability (CV%)**
Sterility	Fungi, aerobic, and anaerobic bacteria	Negative	/
Viral safety	NAT testing (HIV, HBV, HCV, and WNV)	Negative	/
Biochemical analysis	pH	Standard values for human blood	<10%
	Total protein and albumin	Standard values for human blood	<10%
	Hemoglobin	<0.4 g/dl	<10%
Growth factor analysis	Mass spectrometric analysis	/	No. of proteins varied <20%
Culture conditions	Concentration in culture medium	≤10%	<10%
	Heparin concentration	≤2 UI/ml	<10%
Performance testing	Cell proliferation vs. reference FBS and hPL batch using the same reference cells	Population doubling time (PDT) ≤50 h	<10%
Stability	After 6 months of hPL storage at −80°C cell proliferation test (MSC)	Population doubling time (PDT) ≤50 h	<10%
Expiry date	Up to 5 years at −80°C	Annual confirmation of stability after the second year of storage	/

## Discussion

In the near future, regenerative medicine protocols will have to face an increasing demand for hMSC batches to treat patients over a range of diseases, while availability may be limited (Korbling and Estrov, 2003; Sherman et al., 2017). Hence, it will become mandatory to develop hMSC production systems, characterized by higher effectiveness, speed, and reproducibility, in order to address the heightened clinical demand (Le Blanc and Davies, 2018). Nevertheless, hMSC *ex vivo* expansion will need to be accomplished in compliance with GMP guidelines to provide clinically usable cell doses. In this regard, the most relevant role will be played by hPL, since FBS might be a source of xenogenic and immunogenic antigens and zoonotic infections, and therefore, it poses tremendous concerns to regulatory authorities, aside from ethical and economic issues (van der Valk et al., 2018; European Directorate for the Quality of Medicines & HealthCare of the Council of Europe [EDQM], 2020b; European Medicine Agency [EMA], 2020).

From the early 1980s, hPL has been tested as an animal-free culture supplement for cell proliferation (Lucarelli et al., 2003), and it has been subsequently standardized and validated for large-scale clinical grade hMSC production (Doucet et al., 2005; Capelli et al., 2007; Hemeda et al., 2014; Muraglia et al., 2014; Shih and Burnouf, 2015; Strandberg et al., 2017). However, the high batch-to-batch variability remains a reason for further research and laboratory work (Biebak et al., 2019). Similarly, published data (Burnouf et al., 2016) highlight the numerous variables involved in the production of hPL and the resulting challenges in replicating the desired clinical effects (Sherman et al., 2017; Strunk et al., 2018; Biebak et al., 2019; Henschler et al., 2019; Schallmoser et al., 2020). The hPL product is still a subject of debate, due to the too many variables involved in its manufacturing process (Burnouf et al., 2016). AABB and ISCT have established a joint task force charged “to identify gaps of knowledge that could eventually lead to developing and defining recommendations for standardized manufacturing and quality control of hPL” (Biebak et al., 2019). In order to standardize the production of hPL, we have addressed the problem of batch-to-batch variability from the point of view of a blood bank: we have developed a rigorous definition of the starting materials and of the variables in the production process (Table 1), and we have set standards for a ready-to-use product release (Table 2).

Since it is difficult to estimate the real risk of the transmission of anti-HLA antibodies through cell therapy products, we have selected only non-transfused male donors between 18 and 60 years old. To avoid potential immune activation and to limit hemolysis during production, we have assembled group 0 BC resuspended in group AB CPP. While hPL obtained from expired or fresh platelet concentrates has shown a similar proliferative stimulus (Jonsdottir-Buch et al., 2013; Dessels et al., 2019), it has been reported that the composition of growth factors (GF) and cytokines may differ (Sellberg et al., 2016). Since we wanted to limit any unnecessary variables, we only used fractionated blood components within 24 h of collection.

With respect to the number of donors, our policy foresees to pool a total of 25 donors (18 for buffy coats and seven for plasma) per hPL batch. This number has the advantage to reduce donor-related variability, at the same time taking into account the reported concerns for a higher viral contamination risk when using more donors (Stuhler and Blumel, 2015; European Directorate for the Quality of Medicines & HealthCare of the Council of Europe [EDQM], 2020b). A mathematical approach has recently shown that assembling more than 16 donations, in addition to increasing the infection risk, does not improve the composition of GF (Agostini et al., 2017). Our data confirm these findings, by showing that the proliferative effect of pooling together three hPL4c batches (ABC mix: A18 + B18 + C18 equally) is comparable to the effect of each single one (Figure 4B). Moreover, the implementation of pathogen inactivation procedures and/or removal steps constitute a further step that we will have to take to improve safety. Different technologies and strategies based on treatments with solvents/detergents (S/D) or photochemical processes have been described (Schlenke, 2014; Henschler et al., 2019). However, considering that those technologies employ additives or intercalating agents that may be ineffective against some viruses, we will evaluate to implement viral inactivation of hPL through gamma irradiation (minimum dose 35 kGy), which was proven effective without altering growth factor features (Viau et al., 2019).

One of the critical points of hPL production is the procedure for platelet lysis to induce GF release. The available lysis methodologies could influence the GF profiles and, consequently, the performance of the product itself. We have adopted the freeze/thaw technique that is the most used, simple, and non-expensive approach. However, the number of cycles and freeze and thaw temperatures represent further variables that need to be defined (Biebak et al., 2019). Too many freeze/thaw cycles can result in growth factor degradation, whereas an insufficient number of freeze/thaw cycles could affect growth factor concentration due to ineffective platelet breakage (Strunk et al., 2018; Biebak et al., 2019). Since optimal results have been obtained between three and five cycles (Strandberg et al., 2017), we have decided to repeat four cycles of freezing at −80°C followed by thawing at +37°C by using automated and controlled systems routinely used for hemocomponent production. We have validated the efficiency of GF release and reproducibility between batches by comparing MS-proteomic profile and hMSC proliferative effect of hPL1c vs. hPL4c batches. For hPL4c batches, we documented a protein variability less than 20%, while for hPL1c, protein variability resulted higher than 20% (Figure 2B). The results of the hMSC expansion confirmed those findings, showing a more uniform proliferation effect between hPL4c batches than the one existing among hPL1c batches (Figure 4B). Moreover, hPL4c induces a significant decrease of DT in comparison with hPL1c (*p* < 0.001), both at 4 and 7 days of culture (Supplementary Figures 3, 4). The comparison between different preparations might prove useful to decode proteins that drive the growth and differentiation processes in *in vitro* models of regenerative medicine.

A further variable to consider is the medium used for platelet resuspension, storage, and lysis, which can affect cell expansion (Shih and Burnouf, 2015). When plasma is considered as an option (Muraglia et al., 2017), it is mandatory to prevent gelling by heparin addition (Hemeda et al., 2013), the dose of which depends on hPL concentration and fibrinogen content (Burnouf et al., 2016). Our best results were obtained with a fibrinogen reduction >20%, in a medium containing 5% hPL and 0.6 IU/ml of sodium heparin. The chance to minimize heparin addition can affect hMSC expansion and differentiation capabilities (Hemeda et al., 2013), interfere with the CXCR4/SDF-1 signaling pathway (Seeger et al., 2012), and regulate osteoblast metabolism (Hausser and Brenner, 2004). However, the effects of heparin and fibrinogen on hMSC need further insights, in light of the recently published data showing that, despite the differences induced by heparin in gene expression and protein profiles, hMSC characteristics are not affected (Laner-Plamberger et al., 2019).

Last but not least, small variations in the qualitative and quantitative composition of hPL can determine major effects on cultured cells. Therefore, “hPL should be considered a product category and not a single product” in which it is important to distinguish “essential” from “non-essential” content based on the intended application (Henschler et al., 2019). We have previously reported that our hPL4c A18 batch does not induce hMSC chondrogenic differentiation when used alone, but it exerts an inhibitory effect when used in combination with a specific chondrogenic differentiation medium containing TGFβ3 (Manferdini et al., 2019). It has been reported that the composition of hPL can induce hMSC to release cytokines affecting their proliferative and differentiative potential (Mathews et al., 2014; Karlsen and Brinchmann, 2019). Similarly, a study comparing four commercially available hPL has shown that not all of them were able to support hMSC osteogenic differentiation (Boraldi et al., 2018).

## Conclusion

A controlled and standardized process is applicable to the production of hPL, by mimicking the processing of blood products. We have proposed the acceptability criteria of the starting materials, the time and conditions, instruments, and quality/safety requirements of the production together with standards for release. Therefore, we have turned the processing of hPL from a homemade production to a manufacturing process which is comparable and consistent over time. Furthermore, its *off-the shelf* availability in large batches renders hPL no longer a variable factor in hMSC expansion protocols.

Future research must provide answers to several open questions: (1) the identification of growth factors and their correct concentration for a specific cell type and for targeted cell therapy applications; (2) the role of plasma and serum proteins in maintaining cell cultures; (3) the potential role of donor selection (health, age, and sex) on hPL potency and applicability; (4) the need and benefits of fibrinogen depletion and heparin use during cell expansion; (5) the identification of minimum safety and quality standards and the release criteria adequate for the different biological materials and production protocols used; and, last but not least, (6) a revision of the nomenclature of platelet-origin products which would allow a more effective comparison across published data.

## Data Availability Statement

The datasets presented in this study can be found in online repositories. The names of the repository/repositories and accession number(s) can be found below: https://massive.ucsd.edu/ProteoSAFe/dataset.jsp?task=b0ba025c0b654b4abaa60c6042419b64.

## Ethics Statement

The Local Research and Ethics Committee approved the use of human samples in accordance to Helsinki Declaration; blood and bone marrow donors provided written informed consent.

## Author Contributions

AB: conceptualization, methodology, validation, investigation, and writing – original draft. CC: methodology, validation, investigation, visualization, and writing – original draft. MG: validation, formal analysis, data curation, visualization, and writing – original draft. SB, AN, RV, GP, and LP: investigation. GL, FM, and DR: writing – review and editing. CA: conceptualization, writing – original draft, writing – review and editing, and supervision. All authors contributed to the article and approved the submitted version.

## Conflict of Interest

The authors declare that the research was conducted in the absence of any commercial or financial relationships that could be construed as a potential conflict of interest.

## Footnotes

1 www.doubling-time.com

2 https://cran.r-project.org/

## Abbreviations

AABB: American Association of Blood Banks
ANOVA: analysis of variance
BC: buffy coat
BM: bone marrow
hMSC: human mesenchymal stromal cells
CPP: cryoprecipitate-poor plasma
DT: doubling time
FASP: filter-aided sample preparation
FBS: fetal bovine serum
FDR: false discovery rate
GF: growth factors
GMP: good manufacturing practices
GVHD: graft-versus-host disease
HBV: hepatitis B virus
HCV: hepatitis C virus
HIV: human immunodeficiency virus
hPL: human platelet lysate
HSC: high stringency criteria
ISCT: International Society for Cell & Gene Therapy
LSC: low stringency criteria
MS: mass spectrometry
MSC: middle stringency criteria
NAT: nucleic acid test
PLT pool: platelet pool
RBC: red blood cells
SD: standard deviation
TIC: total ion current
WNV: West Nile virus
