# Supplementary material for: A Blood Bank Standardized Production of Human Platelet Lysate for Mesenchymal Stromal Cell Expansion: Proteomic Characterization and Biological Effects
Source: Front Cell Dev Biol. 2021 May 14;9:650490. doi: 10.3389/fcell.2021.650490 (PMC8160451; doi:10.3389/fcell.2021.650490)
Supplement: Supplementary file 1 [file Table_1.pdf]

Supplementary Table 1. Variables in hPL production, as reported in published papers

Variables	Description
Starting material	Plt-apheresis, BC, whole blood
Expired material	Seldom
AB0 group	Undefined
Dilution medium	Plasma, T-sol
Pooling	2-12 units
Platelets count	Mostly undefined
Centrifugation speed	Seldom
Freezing T°	-20°C to -196°C
Filtration	Undefined
Fibrinogen reduction	Rare
Tested cells	BM-, AT-, UCB-hMSC, fibroblast
Comparison to FBS	Rare
Growth factors analysis	Mostly ND

BC: buffy-coat; BM: bone marrow; AT: adipose tissue; UCB: umbilical cord blood; hMSC: human mesenchymal stromal cells; ND: not done.
Sources: Biebak, K., Fernandez-Munoz, B., Pati, S., and Schaefer, R. (2019), Burnouf, T., Strunk, D., Koh, B. M. C., and Schallmoser, K. (2016) and Lucarelli, E., Beccheroni, A., Donati, D., Sangiorgi, L., Cenacchi, A., Del Vento, A. M., et al. (2003).
