# Supplementary material for: A Blood Bank Standardized Production of Human Platelet Lysate for Mesenchymal Stromal Cell Expansion: Proteomic Characterization and Biological Effects
Source: Front Cell Dev Biol. 2021 May 14;9:650490. doi: 10.3389/fcell.2021.650490 (PMC8160451; doi:10.3389/fcell.2021.650490)
Supplement: Supplementary file 2 [file Table_2.pdf]

Supplementary Table 2. Biochemical analysis of the three lots of hPL with 4 freeze/thaw cycles compared to commercial FBS (mix of three lots)

Parameters	hPL4c A18	hPL4c B18	hPL4c C18	hPL4c average	FBS mix
**Glucose (mg/dL)**	319.30	291.50	312.10	307.63	70.10
**Calcium (mg/dL)**	8.40	7.93	8.46	8.26	14.12
**Sodium (mM/L)**	171.60	163.00	172.00	168.87	137.90
**Potassium (mM/L)**	4.71	4.63	4.76	4.70	12.02
**Chloride (mM/L)**	70.30	68.00	70.60	69.63	96.90
**Phosphorus (mg/dL)**	12.72	12.35	12.87	12.65	9.40
**Magnesium (mg/dL)**	0.84	0.79	0.83	0.82	1.31
**Folate (ng/mL)**	3.33	3.56	3.93	3.61	6.49
**Vit B12 (ng/mL)**	308.00	284.00	352.00	314.67	321.00
**Hemoglobin (g/dL)**	0.04	0.03	0.04	0.04	0.02
**Fibrinogen (g/dL)**	0.185	0.173	0.191	0.183	Not Done
**Total proteins (g/dL)**	6.05	5.61	6.13	5.93	3.95
**A/G ratio**	1.54	1.70	1.53	1.59	Not Done
**Albumin (g/dL)**	3.67	3.53	3.70	3.63	Not Done
**α1 Globulin (g/dL)**	0.23	0.21	0.23	0.22	Not Done
**α2 Globulin (g/dL)**	0.49	0.38	0.47	0.45	Not Done
**β1 Globulin (g/dL)**	0.37	0.32	0.37	0.35	Not Done
**β2 Globulin (g/dL)**	0.38	0.33	0.39	0.37	Not Done
**γ Globulin (g/dL)**	0.91	0.84	0.97	0.91	Not Done
**pH**	7.1	7.2	7.2	7.2	7.3
