# Supplementary material for: A Blood Bank Standardized Production of Human Platelet Lysate for Mesenchymal Stromal Cell Expansion: Proteomic Characterization and Biological Effects
Source: Front Cell Dev Biol. 2021 May 14;9:650490. doi: 10.3389/fcell.2021.650490 (PMC8160451; doi:10.3389/fcell.2021.650490)
Supplement: Supplementary file 4 [file Table_4.pdf]

Supplementary Table 4. Summary of the effects of hPL4c and heparin concentrations on hMSC proliferation at 10 days of culture
hMSC batches (hMSC1-2-3 at 4th passage) have been expanded in Iscove's medium (Sigma-Aldrich) containing scalar concentrations of hPL4c (A-B-C18) from 0.6% till 20% and decreasing sodium heparin concentrations from standard 2 IU/mL till heparin-free medium. The average proliferation in the different medium conditions is expressed as Doubling Time in hours (DT) at 10 days of culture. The culture conditions that determined medium gelling are indicated in blue. The culture medium with 5% hPL4c and 0.6 IU/mL of heparin, proved to be the best proliferation condition with the lowest dose of heparin without gelling.
* Average DT of independent experiments (69.37 ±1.96 hours , 67.84±5.76 hours)

Heparin IU/mL in medium						
**2**	NOT PERFORMED	NOT PERFORMED	NOT PERFORMED	80.00	86.30	84.17
**0.6**	NO PROLIFERATION	112.77	86.57	68.61*	71.30	NON-RECOVERABLE CELLS
**free**	NO PROLIFERATION	116.74	89.15	72.48	NON-RECOVERABLE CELLS	NON-RECOVERABLE CELLS
hPL4c % in medium	**0.6**	**1.25**	**2.5**	**5**	**10**	**20**
