# Supplementary material for: A Blood Bank Standardized Production of Human Platelet Lysate for Mesenchymal Stromal Cell Expansion: Proteomic Characterization and Biological Effects
Source: Front Cell Dev Biol. 2021 May 14;9:650490. doi: 10.3389/fcell.2021.650490 (PMC8160451; doi:10.3389/fcell.2021.650490)

Supplementary Figure 1. Blood bank instruments used in hPL production process

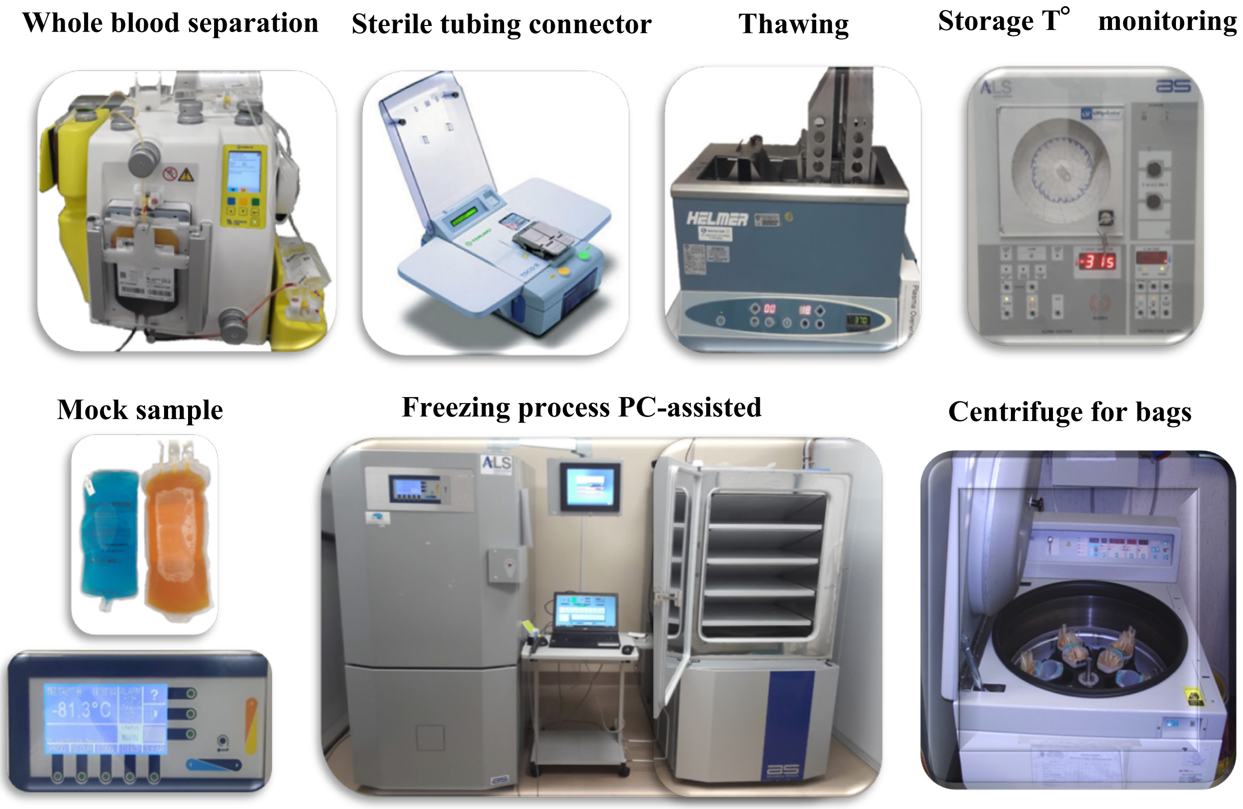

Supplement: Supplementary file 5 [file Image_1.pdf]
