# Supplementary material for: A Blood Bank Standardized Production of Human Platelet Lysate for Mesenchymal Stromal Cell Expansion: Proteomic Characterization and Biological Effects
Source: Front Cell Dev Biol. 2021 May 14;9:650490. doi: 10.3389/fcell.2021.650490 (PMC8160451; doi:10.3389/fcell.2021.650490)

Supplementary Figure 2. hMSC isolation and characterization (immunophenotypic and differentiative)

(**A**) Bone marrow cells are obtained from bag washouts after scheduled donor harvests and stored in liquid nitrogen until use. (**B**) hMSC at passage 2 are characterised for their antigenic profile. hMSC antigenic profile was proved to be positive (>99%) for CD105, CD73 and CD90 and negative for CD45, CD34, CD14 and CD19. (**C**) Confluent hMSC at passage 2 are tested for their differentiation capacity by using specific medium (StemCell Technologies). Briefly, 5 x 10^4 expanded hMSC are detached, washed and cultured with weekly changing of the specific differentiating medium. All cultures were fixed with 4% formaldehyde for 30 min and bone cells were stained with 2% Alizarin Red S solution (pH 4.2) for 3 min, adipocytes with 0.3%, Oil Red O solution for 10 min while chondrocytes micromass was stained with 1% Alcian blue solution in 0.1 N HCl for 30 minutes, to highlight respectively osteogenic, adipogenic and chondrogenic differentiation.

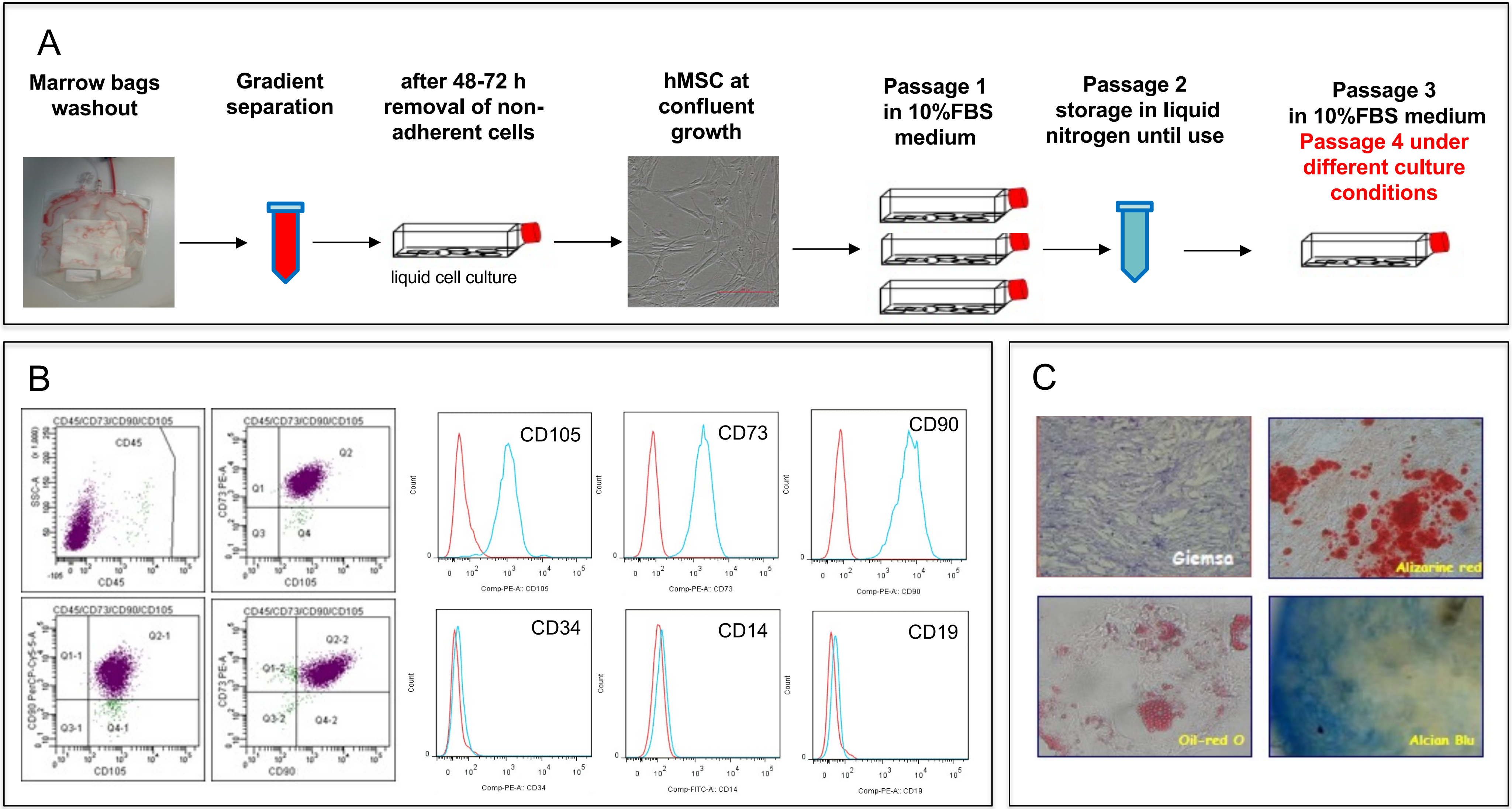

Supplement: Supplementary file 6 [file Image_2.pdf]
