# Supplementary material for: A Blood Bank Standardized Production of Human Platelet Lysate for Mesenchymal Stromal Cell Expansion: Proteomic Characterization and Biological Effects
Source: Front Cell Dev Biol. 2021 May 14;9:650490. doi: 10.3389/fcell.2021.650490 (PMC8160451; doi:10.3389/fcell.2021.650490)

**Supplementary Figure 3. Box-Plot of hMSC expansion at 4 days of culture in presence of FBS versus hPL1c and hPL4c** At starting of expansion assay (day 0), 4000/cm$^2$ hMSC are seeded in presence of 10% FBS mix, 5% of three different batches of hPL1c and hPL4c. Cell proliferation is expressed as Doubling Time (hours). X indicates sample mean. **(A)** Shows the results of all experiments regardless of hMSC and hPL batches used. hPL A17: control-hPL batch previously produced and characterized: (18 donations of BC, 1 freeze/thaw cycle, plasma without removal of fibrinogen, sodium heparin 2 IU/mL). Medians: hPL1c all 40.00, hPL4c all 35.26, FBS mix 63.74, hPL A17 43.98. **(B)** Shows the results for each hPL1c and hPL4c batch.

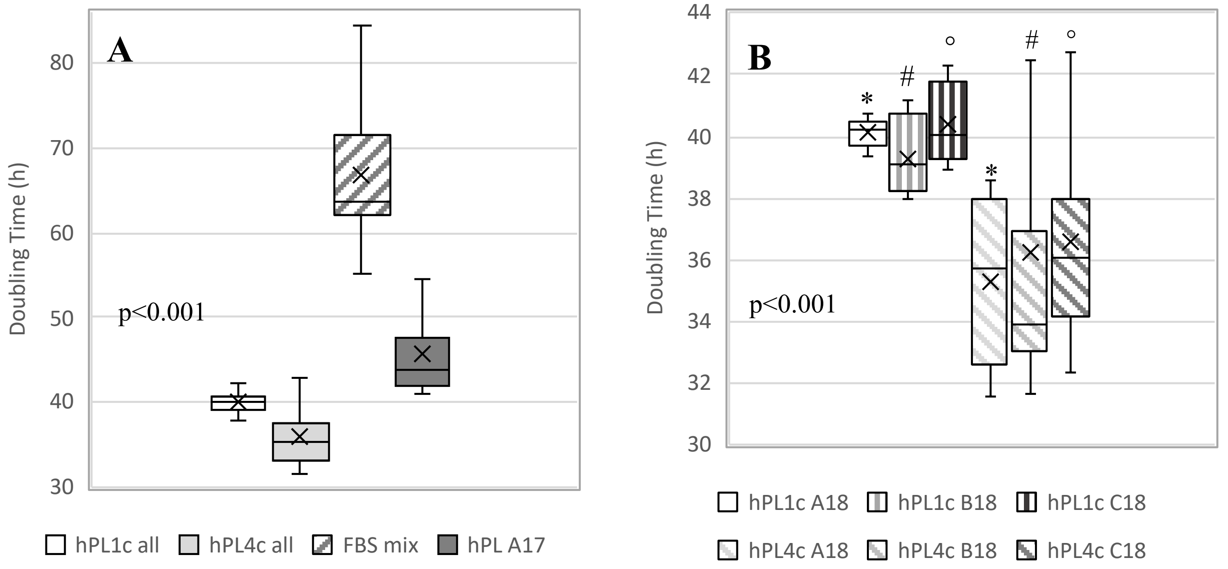

Supplement: Supplementary file 7 [file Image_3.pdf]
