# Supplementary material for: A Blood Bank Standardized Production of Human Platelet Lysate for Mesenchymal Stromal Cell Expansion: Proteomic Characterization and Biological Effects
Source: Front Cell Dev Biol. 2021 May 14;9:650490. doi: 10.3389/fcell.2021.650490 (PMC8160451; doi:10.3389/fcell.2021.650490)
Supplement: Supplementary file 8 [file Image_4.pdf]

Supplementary Figure 4. hPL storage and proliferative effect over time

Box-Plot of hMSC expansion with hPL4c stored at -80 ° C for 6,12 and 24 months from production. Cell proliferation is expressed as Doubling Time (DT in hours) at 7 days of culture. X indicates the mean of the samples. The values refer to the average of the three batches of hPL4c (A-B-C18). Experiments were conducted on 4th passage hMSC2.

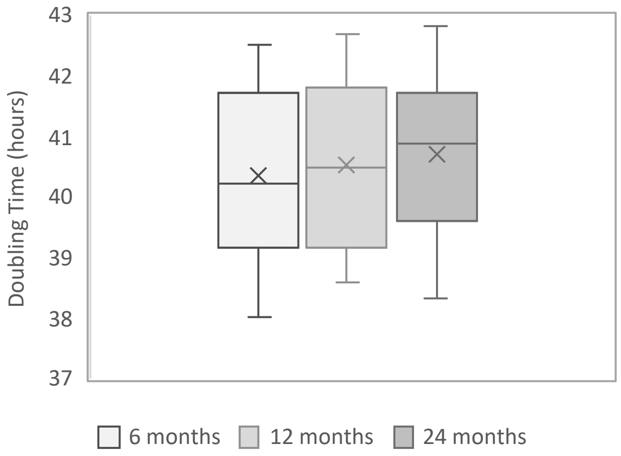

	**6 months**	**12 months**	**24 months**
**mean**	40.20	40.26	40.80
**SD**	1.50	1.16	1.71
**CV%**	3.73	2.89	4.20
